# Nuclear Localization of COX-2 in relation to the Expression of Stemness Markers in Urinary Bladder Cancer

**DOI:** 10.1155/2012/165879

**Published:** 2012-04-10

**Authors:** Raynoo Thanan, Mariko Murata, Ning Ma, Olfat Hammam, Mohamed Wishahi, Tarek El Leithy, Yusuke Hiraku, Shinji Oikawa, Shosuke Kawanishi

**Affiliations:** ^1^Faculty of Pharmaceutical Sciences, Suzuka University of Medical Science, 3500-3 Minamitamagaki-cho, Suzuka, Mie 513-8670, Japan; ^2^Department of Environmental and Molecular Medicine, Mie University Graduate School of Medicine, Tsu, Mie 514-8507, Japan; ^3^Faculty of Health Science, Suzuka University of Medical Science, Suzuka, Mie 510-0293, Japan; ^4^Departments of Pathology and Urology, Theodor Bilharz Research Institute, Giza, Egypt

## Abstract

Inflammation may activate stem cells via prostaglandin E2 (PGE2) production mediated by cyclooxygenase-2 (COX-2) expression. We performed an immunohistochemical analysis of the expression of stemness markers (Oct3/4 and CD44v6) and COX-2 in urinary bladder tissues obtained from cystitis and cancer patients with and without *Schistosoma haematobium* infections. Immunoreactivity to Oct3/4 was significantly higher in *S. haematobium*-associated cystitis and cancer tissues than in normal tissues. CD44v6 expression was significantly higher in bladder cancer without *S. haematobium* than in normal tissues. COX-2 was located in the cytoplasmic membrane, cytoplasm, and nucleus of the cancer cells. Interestingly, the nuclear localization of COX-2, which was reported to function as a transcription factor, was significantly associated with the upregulation of Oct3/4 and CD44v6 in bladder cancer tissues with and without *S. haematobium* infection, respectively. COX-2 activation may be involved in inflammation-mediated stem cell proliferation/differentiation in urinary bladder carcinogenesis.

## 1. Introduction

Chronic inflammation, in which large amounts of reactive oxygen/nitrogen species (ROS/RNS) and cytokines are produced, is a well-recognized cause of cancer [1]. Epidemiologic and animal studies have indicated chronic inflammation including urinary tract infections to be involved in the development of bladder cancer [2, 3]. Infections by parasites such as *Schistosoma haematobium* (*S. haematobium*), which act as inflammatory agents, are associated with urinary bladder cancer [4]. One mechanism of carcinogenesis involves the eggs of parasites [5, 6]. The deposition of parasite eggs in the host bladder results in irritation, eventual fibrosis, and chronic cystitis, leading to carcinogenesis [6]. In areas where *S. haematobium *is not endemic, sporadic bladder cancer may be caused by environment factors, such as smoking [7, 8], and genetic polymorphisms in tumor suppressor [9–11], drug metabolism [12], and anti-oxidant [13] genes.

Recently, evidence is accumulating to show that stem cells are involved in inflammation-related carcinogenesis. According to the cancer stem cell hypothesis, not all tumor cells can participate in tumor evolution, and instead, this property is limited to a subset of cells, termed “cancer stem cells” [14, 15]. Several reports have suggested Oct3/4 to play roles in tumor progression and regulation of the motility of bladder cancer cells [16, 17]. Bentivegna et al. reported the existence of cancer stem cell-like populations in bladder cancer patients, using markers including Oct3/4 [18]. Our recent report demonstrated that Oct3/4 was expressed not only in bladder cancer but also in cystitis tissues in *S. haematobium*-infected patients [19], suggesting a property of stem cell. CD44 is a receptor for hyaluronic acid, and its variant isoform 6 (CD44v6) is a molecular marker for bladder cancer [20]. Several reports have showed that loss of CD44v6 expression in urinary bladder cancer is associated with tumor dedifferentiation, abnormal DNA content, and a poor prognosis [20–22]. However, others reported that CD44v6 was associated with cell migration, tumor growth, and progression, and the expression of CD44v6 is correlated with the proliferation of poorly differentiated urothelial cells [23–25]. These controversial results were reported in previous papers on the relationship between CD44v6 expression and the biological behavior of urothelial cells. Interestingly, recent reports suggested that CD44v6-positive cells possessed a characteristic phenotype of tumor-initiating cancer stem cell in brain tumor [26] and bladder cancer [23, 25]. These reports let us hypothesize that Oct3/4 and CD44v6 may be involved in establishing a stem-like cell phenotype in bladder cancer.

Prostaglandin E2 (PGE2) has been reported to regulate vertebrate hematopoietic stem cell homeostasis [27]. Inflammation may also activate normal or cancer stem cells via PEG2 signaling [28]. It is accepted that cyclooxygenase-2 (COX-2), the PGE2-generating enzyme, is important to inflammation-related carcinogenesis. Using rat urinary bladder carcinogenesis induced by *N*-butyl-4-*N*-(4-hydroxybutyl) nitrosamine (BBN) as a model, the increased expression of the COX-2 protein was found in preneoplastic and neoplastic lesions of tumor tissues [29], and COX inhibitors exhibited antitumor activity [30]. Expression of COX-2 had been demonstrated in transitional cell [31] and squamous cell [32] carcinomas of urinary bladder and *S. haematobium*-associated urinary bladder cancer patients [33]. Therefore, we have assumed that COX-2 plays important roles in not only tumor initiation/promotion but also in the regulation of stem cell proliferation and differentiation in inflammation-related urinary bladder carcinogenesis.

To understand carcinogenic mechanisms in relation to stem cell activation during inflammation, we examined the expression of COX-2 and stemness markers (Oct3/4 and CD44v6) in bladder tissues obtained from cystitis and cancer patients infected with *S. haematobium,* in comparison to that in normal tissues. 

## 2. Materials and Methods

### 2.1. Patients

Formalin-fixed and paraffin-embedded biopsy and surgical specimens were obtained from 33 cases of bladder cancer associated with *S. haematobium *infections (9 cases of squamous cell carcinoma (SCC), 23 cases of transitional cell carcinoma (TCC) and 1 case of adenocarcinoma) and 12 cases of cystitis induced by *S. haematobium *from the Theodor Bilharz Research Institute in Egypt. We assessed pathological grades of urinary bladder cancer according to the World Health Organization Classification [34, 35] as shown in Table 1. *S. haematobium *egg antigens in sera were detected by Sandwich ELISA [36]. This study was performed in accordance with the Ethical Guidelines for Epidemiological Research enacted by the Japanese government. We removed identifying information from all samples before analysis.

Urinary bladder cancer tissus caused by other risk factors (no *S. haematobium *infection, 1 case of SCC, 2 cases of adenocarcinoma, and 37 cases of TCC) and normal tissues adjacent to tumors (31 cases) were obtained from a commercial urinary bladder tissue array (Cat.No.BL801, Biomax.us, USA). Normal tissues with cystitis were excluded.

### 2.2. Immunofluorescence Analysis

Immunoreactivity to COX-2, Oct3/4, and CD44v6 in bladder tissues was assessed by single or double immunofluorescence labeling, as described previously [37]. Briefly, deparaffinized and dehydrated sections (6-*μ*m thick) were incubated with 5% skim milk and incubated with a PBS-diluted antibody (mouse monoclonal anti-Oct3/4 (1 : 50, Santa Cruz Biotechnology), mouse monoclonal anti-CD44v6 (1 : 100, Abcam), or rabbit polyclonal anti-COX-2 (1 : 200, Calbiochem)) overnight at room temperature. The sections were incubated for 3 hours with Alexa 594-labeled goat antibody against rabbit IgG or Alexa 488-labeled goat antibody against mouse IgG (1 : 400) (Molecular Probes, Eugene, Oregon, USA). The stained sections were examined under a confocal laser scanning microscope (FV-1000, Olympus, Tokyo, Japan) or fluorescence microscopy (BX53, Olympus, Tokyo, Japan).

### 2.3. Immunohistochemical Grading

 We defined immunohistochemical grading (IHC grading) based on intensity and frequency derived from the staining results. The staining intensity was scored as negative (0), weak (+1), moderate (+2), or strong (+3). The frequency of positive cells in a section was scored as negative (0), less than 25% (+1), 25–50% (+2), 51–75% (+3), or more than 75% (+4). IHC grading was assigned by multiplying the intensity score by the frequency score, as follows: −, absent expression (0); +, weak expression (1–4), ++, moderate expression (5–8); +++, high expression (9–12).

### 2.4. Statistical Analysis

The significance of differences was determined by Chi-square test. *P* < 0.05 was considered to be statistically significant. The statistical analysis was performed using SPSS19 for Windows.

## 3. Results

### 3.1. Expression of COX-2 in Urinary Bladder Tissues

**Figure 1:**
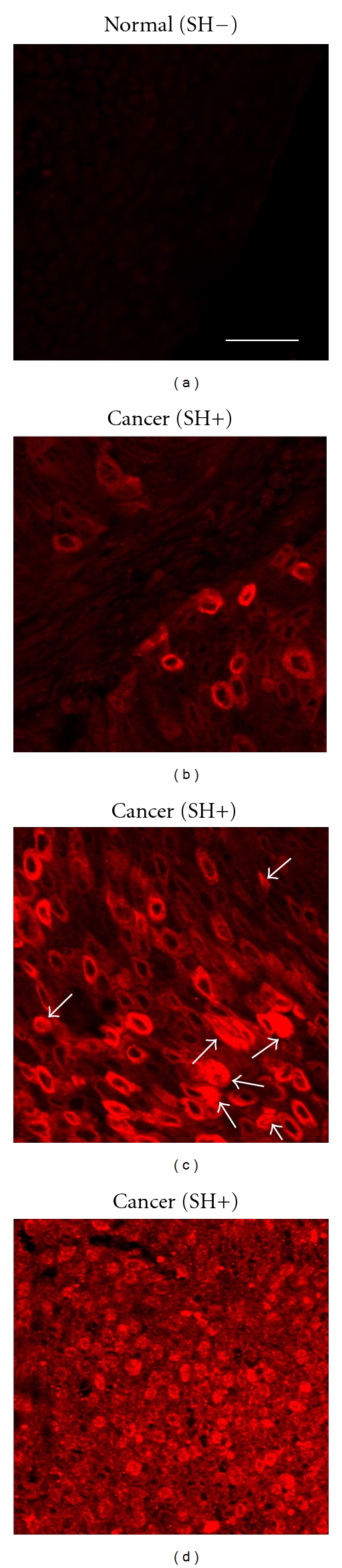
Localization of COX-2 in urinary bladder cancer. COX-2 (red) was stained by an immunofluorescence method in urinary bladder tissues including normal (SH−) and cancer (SH+) tissues. (a) COX-2 was very weakly expressed in normal tissues. Scale bar is equal to 20 *μ*m. (b) COX-2 staining was observed only in the cytoplasm of cancer cells. (c) COX-2 was strongly expressed in both the cytoplasm and nucleus of cancer cells. Arrows indicate nuclear positive staining. (d) COX-2 was strongly expressed in the nucleus of cancer cells. SH: *S. haematobium* infection.

 COX-2 was detected in the plasma membrane, cytoplasm, and nucleus in hyperplasia and precancerous and cancer cells. COX-2 was expressed very weakly in normal urinary bladder tissues (Figure 1(a)). *S. haematobium*-associated urinary bladder cancer tissue had strong expression of COX-2 in only cytoplasm of cancer cells (Figure 1(b)), which was described as a cytoplasmic staining.  Figure 1(c) shows the COX-2 staining in both the cytoplasm and nucleus, while Figure 1(d) represents strong expression of COX-2 in nucleus of the cancer cells. We assigned the patterns shown in Figures 1(c) and 1(d), as positive for nuclear localization of COX-2.  Table 2 summarizes the results of COX-2 expression in urinary bladder tissues. COX-2 staining was observed in 16% (5/31) of normal bladder samples. The expression of COX-2 was significantly increased in 90% (36/40) of bladder cancer patients without *S. haematobium* infections (*P* < 0.001 compared to normal tissues). It was also detected in 61% (20/33) of bladder cancer patients and 58% (7/12) of cystitis patients infected with *S. haematobium* (*P* < 0.001 and *P* = 0.006, resp., compared to normal tissues).

### 3.2. Expression of Oct3/4 and COX-2 in Urinary Bladder Tissues

**Figure 2:**
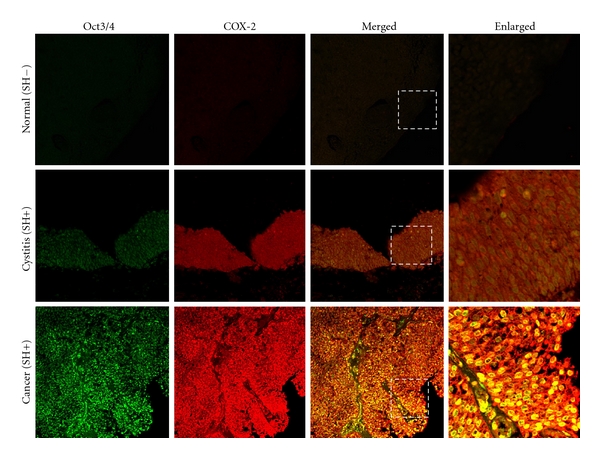
Colocalization of Oct3/4 and COX-2 in urinary bladder samples. The distribution of Oct3/4 (green) and COX-2 (red) was determined by double immunofluorescence in urinary bladder tissues from normal, *S. haematobium*-associated cystitis and cancer subjects. Original magnification is 200x. Enlarged: enlargement of dotted areas. SH: *S. haematobium* infection.

The expression of Oct3/4 and COX-2 in urinary bladder tissues is shown in Figure 2. Oct3/4 was weakly stained in normal epithelium cells. The mucosal layer and precancerous area in cystitis patients with *S. haematobium *infections exhibited strong Oct3/4 signals in nuclei of the mucosa, as well as in urothelial epithelium with glandular metaplasia. The stem cell marker was also strongly expressed in the nucleus of bladder cancer cells of patients infected with *S. haematobium*. Conversely, cancer tissues without *S. haematobium* infections showed weak immunoreactivity to Oct3/4 (data not shown). As summarized in Table 2, immunoreactivity to Oct3/4 was significantly higher in *S. haematobium*-associated cystitis and cancer tissues than normal tissues (*P* = 0.031 and *P* = 0.010, resp.). The expression of Oct3/4 was significantly higher in cancer tissues with the infection than without (*P* < 0.001). Interestingly, the tumor tissues of patients infected with *S. haematobium *exhibited strong colocalization of Oct3/4 and COX-2 in cancer cell nuclei.

### 3.3. Expression of CD44v6 and COX-2 in Urinary Bladder Tissues

**Figure 3:**
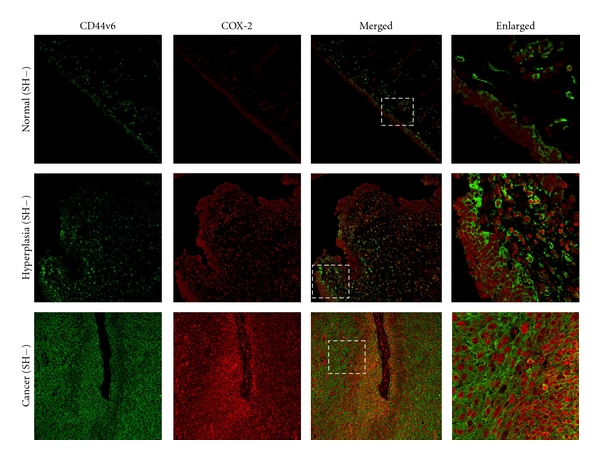
Localization of CD44v6 and COX-2 in urinary bladder samples. The distribution of CD44v6 (green) and COX-2 (red) was determined by double immunofluorescence in normal tissue, hyperplasic tissue nearby tumor, and cancer of urinary bladder tissue. These subjects had other risk factors without *S. haematobium* infections. Original magnification is 200x. Enlarged: enlargement of dotted areas. SH: *S. haematobium *infection.

Figure 3 shows the localization of CD44v6 and COX-2 in bladder tissues without *S.haematobium* infection. CD44v6 localized primarily to the cell membrane, and also to the nuclear membrane. CD44v6 expression was observed at the basal layer of mucosal cells in normal bladder tissues. CD44v6 was also stained in the transitional (mucosal) and precancerous cells of tissues in infected cystitis tissues. Interestingly, most cells from hyperplasia areas and cancers without *S. haematobium* (cancer (SH−)) expressed CD44v6, whereas, the cells from cancers with the parasite (cancer (SH+)) expressed less CD44v6. As shown in Table 2, CD44v6 expression was significantly higher in bladder cancer without *S. haematobium *than in normal tissues without the infection (*P* < 0.001). No significant increase was observed in *S.-haematobium*-associated cystitis (*P* = 0.496) or urinary bladder cancer (*P* = 0.484) compared with normal tissues. Moreover, the immunoreactivity of the stemness marker was significantly higher in urinary bladder cancer tissues without *S. haematobium* infection than in the *S. haematobium*-associated cancer tissues (*P* < 0.001).

### 3.4. Nuclear Localization of COX-2 in Urinary Bladder Cancer

Table 3 shows COX-2 expression in relation to the expression of stemness markers in urinary bladder cancer. The expression of Oct3/4 in *S. haematobium*-associated urinary bladder cancer tissues tended to increase in the patients with COX-2 expression (*P* = 0.060). Interestingly, a significant association was observed between the up-regulation of Oct3/4 and nuclear localization of COX-2 in bladder cancer tissues from patients infected with *S. haematobium* (*P* = 0.001). By contrast, the upregulation of CD44v6 was significantly associated with the expression of COX-2 in urinary bladder cancer patients without the infection (*P* = 0.002). The nuclear localization of COX-2 was more strongly associated with CD44v6 expression (*P* < 0.001).

## 4. Discussion

Inflammation is a well-recognized cause of cancer [38]. However, cancer itself can cause inflammation through the production of proinflammatory factors such as NF-*κ*B, matrix metalloproteases (MMPs), inflammatory cytokines, and chemokines [38, 39]. Inflammation-associated tissue injury may activate stem cells [40, 41]. PGE2 has been reported to function as a potent regulator of hematopoietic stem cell differentiation, homing, survival, and proliferation [27, 42]. In mouse embryonic stem cells, PGE2 also plays a role in the induction of proliferation and suppression of apoptosis [43, 44]. Moreover, PGE2 is found to induce human mesenchymal stem cell migration and proliferation mediated by interaction of profilin-1 and F-actin via a beta-arrestin-1/JNK signaling pathway [45]. Rudnick and co-workers demonstrated that fibroblasts secreting abundant amounts of PGE2 were capable of enhancing tumor growth *in vivo* and could increase the number of cancer stem-like cells [46]. These results suggest that PGE2 plays a crucial role in the regulation of several types of stem cells. Many cancers including bladder cancer exhibit high PGE2 levels due to upregulated expression of COX-2, a key enzyme in PGE2 biosynthesis [31–33]. It is accepted that COX-2 is important to inflammation-related carcinogenesis. Therefore, inflammation may also activate normal or cancer stem cells via PEG2 signaling [28]. The COX-2 enzyme is generally expressed in the cytoplasm. However, studies have indicated that its accumulation in the nucleus was indicative of a functional role as a transcription factor in regulating gene expression [47, 48]. Here, we found the nuclear localization of COX-2 to be significantly correlated with expression of the stemness markers Oct3/4 and CD44v6 in *S. haematobium*-associated urinary bladder cancer and cancer tissues without infection, respectively. These results suggest that the activation of COX-2 in urinary bladder carcinogenesis not only generates PGE2 but also may be involved in the regulation of Oct3/4 and CD44v6 expression.

Oct3/4, a member of the family of POU-domain transcription factors, is generally expressed in pluripotent embryonic stem and germ cells [49, 50]. The embryonic stem cell marker has been reported to be involved in the reprogramming of mature cells into germline-component-induced pluripotent stem cells [51–53]. Recently, it is suggested that Oct3/4 is also a cancer stem cell marker in various types of tumors including urinary bladder cancer [17]. Expression of Oct3/4 has been reported necessary for maintaining the self-renewing, cancer stem-like, and chemoradioresistant properties of tumorigenic stem-like cell populations [54, 55]. In addition, Oct3/4 has been reported to play roles in tumor progression and regulation of the motility of bladder cancer cells [16, 17]. The present study indicated that the expression of Oct3/4 was significantly increased in *S. haematobium*-associated cystitis and cancer tissues compared with normal tissues. Therefore, Oct3/4 could be a useful stemness marker for parasite-related carcinogenesis in relation to the differentiation/reprogramming of stem-like cells.

CD44 has been identified as a cell surface marker associated with cancer stem cells in several types of tumors including urinary bladder cancer [20]. Numerous isoforms of CD44 are generated through alternative mRNA splicing [56]. Variant isoforms (CD44v) with insertions in the membrane-proximal extracellular region are abundant in epithelial-type carcinomas and were found to be associated with the progression of gastrointestinal malignancies [57]. Both CD44- and CD44v-positive stem cell-like phenotypes have been reported to have high-capacity antioxidant systems rich in glutathione that result in growth arrest, cell differentiation, and senescence and thereby promote the proliferation of cancer cells [58, 59]. Moreover, expression of CD44v6 is correlated with proliferation of poorly differentiated urothelial cells and the characteristic phenotype of tumor-initiating bladder cancer stem cells [23, 25]. In this study, we also showed that CD44v6 was expressed in the mucosa basal layer of normal and hyperplasia tissues, and the expression was significantly increased in bladder cancer tissues compared to normal without the parasite. Therefore, CD44v6 may have a relationship with sporadic bladder cancer in the absence of the infection.

The present study demonstrates that different risk factors induce different levels of expression of stemness markers in urinary bladder carcinoma. *S. haematobium*-induced urinary bladder cancer correlates with the expression of Oct3/4, while the cancer without the infection correlates with the expression of CD44v6. The expression of both stemness markers was significantly correlated with the nuclear localization of COX-2, suggesting that inflammation might mediate stem cell proliferation and differentiation/reprogramming in urinary bladder cancer.

## Figures and Tables

**Table 1:** Pathological analysis of urinary bladder tumor tissues.

Risk factor	Pathology diagnosis	Tumor grade	Case
*S. haematobium *infection (SH+)	Transitional cell carcinoma	Well differentiated	4
		Moderately differentiated	11
		Poorly differentiated	8
		
	Squamous cell carcinoma	Well differentiated	2
		Moderately differentiated	5
		Poorly differentiated	2
		
	Adenocarcinoma	Moderately differentiated	1

No *S. haematobium *infection (SH−)	Transitional cell carcinoma	Well differentiated	8
		Moderately differentiated	16
		Poorly differentiated	13
		
	Squamous cell carcinoma	Moderately differentiated	1
		
	Adenocarcinoma	Moderately differentiated	1
		Poorly differentiated	1

**Table:** (a) COX-2

^$^COX-2 staining	−	*+*	*P value
Normal (SH−)	26	5	
Cancer (SH−)	4	36	<0.001^a^
Cystitis (SH+)	5	7	0.006^a^
Cancer (SH+)	13	20	<0.001^a^

**Table:** (b) Oct3/4

^#^IHC grading	−	*+*	*++*	*+++*	*P* value	
Normal (SH−)	19	11	0	1		
Cancer (SH−)	30	10	0	0	0.299^a^	
Cystitis (SH+)	3	7	2	0	0.031^a^	
Cancer (SH+)	8	17	2	6	0.010^a^	<0.001^b^

**Table:** (c) CD44v6

IHC grading	−	*+*	*++*	*+++*	*P* value	
Normal (SH−)	14	14	2	1		
Cancer (SH−)	3	11	15	11	<0.001^a^	
Cystitis (SH+)	6	3	2	1	0.496^a^	
Cancer (SH+)	19	9	4	1	0.484^a^	<0.001^b^

^$^COX-2 staining results were assigned as (−) negative and (+) positive staining.

^#^IHC grade was assigned to each specimen according to the degree of staining as described in Materials and Methods.

SH: *S. haematobium *infection.

*P values were calculated by Chi-square test. ^a^Versus normal (SH−). ^b^Versus cancer (SH−).

**Table 3:** Expression of Oct3/4 and CD44v6 in urinary bladder cancer patients positive and negative for COX-2 expression.

Risk factor	Stemness marker	^#^IHC grading	COX-2 expression		Nuclear localization of COX-2	
			Negative case	Positive case	Negative case	Positive case
*S. haematobium *infection (SH+)	Oct3/4	−	5	3	8	0
		+	8	9	10	7
		++	0	2	0	2
		+++	0	6	0	6
		*P-value	*P* = 0.060		*P* = 0.001	
	CD44v6	−	9	10	10	9
		+	2	7	5	4
		++	1	3	2	2
		+++	1	0	1	0
		*P*-value	*P* = 0.320		*P* = 0.826	

No *S. haematobium *infection (SH−)	Oct3/4	−	3	27	4	26
		+	1	9	2	8
		++	0	0	0	0
		+++	0	0	0	0
		*P*-value	*P*=1.00		*P* = 0.609	
	CD44v6	−	2	1	3	0
		+	2	9	3	8
		++	0	15	0	15
		+++	0	11	0	11
		*P*-value	*P* = 0.002		*P* < 0.001	

^#^IHC grade was assigned to each specimen according to the degree of staining as described in Materials and Methods.

SH: *S. haematobium *infection.

*P values were calculated by Chi-square test.
